# Adherence of physicians to evidence-based management guidelines for treating type 2 diabetes and atherosclerotic cardiovascular disease in Ajman, United Arab Emirates

**DOI:** 10.1186/s12875-022-01672-4

**Published:** 2022-04-07

**Authors:** Farah Jabbar Ali Alliabi, Ammar Ali Saleh Jaber, Mahir Khalil Ibrahim Jallo, Mirza R. Baig

**Affiliations:** 1 Department of Clinical Pharmacy & Pharmacotherapeutics, Dubai Pharmacy College for Girls, Po Box 19099, Al Muhaisanah 1, Al Mizhar, Dubai, UAE; 2 Clinical Professor of Medicine - Department of Clinical Sciences - College of Medicine - Gulf Medical University, Thumbay University Hospital - UAE, P.O. Box 22695, Al Wahda, Sharjah, UAE

**Keywords:** Type II diabetes mellitus, Treatment guidelines, Physicians’ adherence, Atherosclerotic cardiovascular disease, Body mass index, Hemoglobin A1c, Estimated average glucose

## Abstract

**Background:**

Good adherence by physicians to treatment guidelines for type II diabetes mellitus (T2DM) could improve therapy outcome for patients. In this retrospective, cross-sectional study, we assessed physicians’ adherence to evidence-based guidelines for T2DM management in adult patients (aged ≥18 years) with either confirmed atherosclerotic cardiovascular disease (ASCVD) or those at high risk of developing ASCVD at the Thumbay Academic Health Center, United Arab Emirates (UAE).

**Methods:**

Relevant data was obtained from patients’ medical records, assessed, and compared based on the 2018 diabetes guidelines of the American Diabetes Association and European Association for the Study of Diabetes.

**Results:**

A total of 218 patients (186 males and 32 females) were included in the analysis. Of these, 122 were prescribed either sodium-glucose co-transporter-2(SGLT2) inhibitors or glucagon-like peptide 1 (GLP-1) receptor agonists and 34 were prescribed both. The overall adherence to the guidelines was 56%, which was significantly influenced by body mass index (BMI), hemoglobin A1c (HbA1c) levels, and estimated average glucose (eAG).

**Conclusions:**

Adherence to guidelines was significantly high when treating patients with elevated levels of HbA1c and eAG, suggesting that physicians are more likely to prescribe SGLT2 inhibitors or/and GLP-1 receptor agonists to such patients. Physicians’ adherence to guidelines was significantly correlated with patients’ BMI and the levels of HbA1c and eAG. To the best of our knowledge, this is the first study conducted on diabetes and its risk factors in UAE.

## Background

The burden of diabetes is increasing worldwide, with the main contributing factors being poor nutrition and physical inactivity. Globally, the number of adult patients with diabetes has increased from 108 million in 1980 to 422 million in 2014 (2019) [1]. The International Diabetes Federation (IDF) reported that in 2017, 17.3% of the population of the United Arab Emirates (UAE) aged between 20 and 79 years had type II diabetes mellitus (T2DM). To date, UAE ranks 15th in the global list of countries in terms of age-adjusted comparative prevalence of diabetes with 1 million diabetic individuals [2]. This disorder exerts a substantial economic burden on the country, owing to costs associated with unemployment, productivity loss, early patient death, and increased health service demands [2, 3]. Therefore, early intervention and optimal care provision are crucial to reducing the prevalence of diabetes and its associated risk factors in the UAE.

Most patients with T2DM have additional risk factors, such as dyslipidaemia, hypertension, obesity, chronic kidney disease (CKD), physical inactivity, and smoking habits; however, atherosclerotic cardiovascular disease (ASCVD) is the primary cause of mortality in patients with T2DM [4]. Furthermore, deaths associated with ASCVD events are reported when multiple ASCVD risk factors occur concurrently [5, 6]; therefore, managing modifiable ASCVD risk factors is beneficial for patients with diabetes [7]. Although the implementation of evidence-based approaches has significantly reduced ASCVD events and mortality rates in patients with diabetes [7], educating physicians on the benefits of a multi-factorial approach for T2DM management, including ASCVD risk control, remains essential [4].

Cost-effective screening procedures, treatment guidelines, and diagnostic and curative approaches have been shown to influence the treatment outcomes of patients with T2DM positively [8]. The current standard of care recommendations, such as the Standards of Medical Care in Diabetes approved by the American Diabetes Association (ADA), consider patients’ preferences and comorbidities, among other factors [3, 9]. The ADA regularly updates their standards to ensure optimum care for patients with diabetes [4]. Furthermore, the Diabetes Canada Clinical Practice Guidelines (DCCPG) helps guide clinical practice, disseminate knowledge on the basic modes of management, improve diabetes prevention trials in Canada, and minimise the burden of diabetes and its complications [10]. In 2018, the DCCPG was updated to contain well-revised diagnostic, prognostic, and therapeutic recommendations for the management and prevention of onset of diabetes. Although the updated guidelines were initially intended to enhance the standard of care for patients with diabetes in Canada, they can also be applied to improve diabetes care globally, including the UAE, to address gaps in clinical care and discrepancies between the current evidence-based science and clinical practice [10].

An international panel that included patients, clinicians, and methodologists introduced risk stratified recommendations and guidelines using standards for trustworthy guidelines and the GRADE approach concerning the prescription of GLP-1 receptor agonists and SGLT2 inhibitors [11]. The treatment guidelines clearly state weak recommendations in a number of scenarios: against starting GLP-1 receptor agonists or SGLT2 inhibitors for patients with three or fewer cardiovascular risk factors without established cardiovascular disease (CVD) or chronic kidney disease (CKD); starting SGLT2 inhibitors and against starting GLP-1 receptor agonists for patients with more than three cardiovascular risk factors without established CVD or CKD; for starting SGLT2 inhibitors and GLP-1 receptor agonists; for patients with established CVD or CKD. Strong recommendation is stated in the following scenarios: for starting SGLT2 inhibitors and weak recommendation for starting GLP-1 receptor agonists for patients with established CVD and CKD; and weak recommendation for starting SGLT2 inhibitors rather than GLP-1 receptor agonists for patients committed to further reducing their risk for CVD and CKD outcomes. These guidelines were applied as the basis for assessing physicians’ adherence.

In this study, we assessed the adherence of physicians to the 2018 Clinical Practice Guidelines for the management of T2DM in patients in the UAE, with either confirmed ASCVD or those at high risk of developing ASCVD. The factors that influence the physicians’ adherence to treatment guidelines were also evaluated. These objectives are imperative, as assessing the current standard of care in the private sector in the Emirate of Ajman against the requirements outlined in the 2018 Clinical Practice Guidelines will help ensure that the private facilities in the UAE meet all the required guidelines for optimum patient care.

## Methods

### Study design

This was a retrospective cross-sectional study conducted in outpatient clinics and in the in-patient wards at the Thumbay Academic Health Centre in the UAE from 1 January 2018 to 30 December 2019. The patients collected their medication on a weekly basis. About 75% of the hospital patients have Medical insurance with different categories of cover. Few of the insurance are basic with restricted annual budget for medications which definitely impacted the prescription choice. The remaining 25% of patients are self-pay with different socioeconomic capabilities to pay for all the prescribed medications specially the costly GLP1 & SGLT2I medications.

### Inclusion/exclusion criteria

The study group comprised patients with T2DM (aged ≥18 years) with either confirmed or at high risk of ASCVD who visited the outpatient diabetic clinic or were admitted in the in-patient wards of the health centre during the study. This group is defined as the high-risk population.

### Sample size calculation

OpenEpi was used to calculate the sample size for this study (https://www.openepi.com/SampleSize/SSCohort.html) at a significance level of 5%, confidence level of 95%, and an assumption of 50% adherence of physicians to the 2018 Clinical Practice Guidelines. The alpha level (α) was set at 5% to have a 95% confidence interval (CI). Precision (D) of the 95% CI was set at 5% to ensure that the width of the 95% CI will be maximum 10%. Based on the above assumptions, a sample size (n) of at least 384 participants was required for the study.

### Data collection

Anthropometric, biochemical, and all other related data were obtained from the medical records of patients with T2DM, registered in the outpatient and in-patient wards at the Thumbay Academic Health Centre. The physicians include consultants in internal medicine, diabetes and endocrinology. The patients in the study are those patients with diabetes and established cardiovascular disease or very high risk. They are under the care of cardiologist and endocrinologist in the hospital. Still good number of the patients without established cardiovascular disease with high risk under internist care, being not referred from internist to endocrinologist or cardiologist as should be. This research was impacted the hospital policy for proper patient referral. Being a tertiary care hospital, no primary care physicians in the hospital decided the research patient prescription. The data included socio-demographic information, such as age, sex, and clinical characteristics, including body mass index (BMI), blood pressure, T2DM duration, current treatment, and social history (smoking and alcohol consumption). Laboratory results were obtained from patient files and included the levels of hemoglobin A1c (HbA1c), fasting blood sugar (FBS), total cholesterol (TC), triglyceride (TG), high-density lipoprotein cholesterol (HDL-C), low-density lipoprotein cholesterol (LDL-C), and average glucose (eAG). Relevant data were assessed and compared, based on the updated 2018 Diabetes Guidelines (ADA/EASD Guidelines).

### Ethical considerations

Patient confidentiality was maintained throughout the study. Data extracted from patient files did not link to their names, which were replaced with codes to represent each patient. The Thumbay Hospital Ajman, the academic training centre of Gulf Medical University is one of the big private health care providers in Northern Emirates in UAE. As any private company in UAE, not all patients included in the study were reimbursed for the medications. This will prevent bias in the responses.

### Statistical analysis

Data were analysed using Statistical Package for Social Sciences (SPSS, version 26) and examined for data entry errors and outlying values. The physicians’ adherence to the guidelines of diabetic management was assessed based on the prescription of SGLT2 inhibitors (empagliflozin, dapagliflozin, dapagliflozin 5 mg/metformin 500 mg and empagliflozin /metformin) or GLP-1 receptor agonists (liraglutide, dulaglutide and insulin degludec 100 IU/mL/liraglutide, 100 IU/ml/Lixisenatide Liraglutide (Victoza), lixisenatide and tresiba) to patients with either confirmed or at high risk of ASCVD. The level of physicians’ adherence, based on patients’ biochemical markers was also assessed. Data are presented as the mean with standard deviation (SD) for quantitative variables and frequency and as percentages for categorical variables. Physicians’ adherence to the new guidelines for the management of T2DM is expressed as a percentage, with 95% CI. Several variables were assessed for statistical significance using the chi-squared test. Univariate and multivariate logistic regression analyses were used to examine factors associated with physicians’ adherence to the new guidelines. A *P* value <0.05 was considered significant.

## Results

### Baseline and anthropometric characteristics of T2DM patients with confirmed or at high risk of ASCVD

218 (56.7%) met the inclusion criteria (186 males and 32 females). The baseline and anthropometric data of the patients are presented in Table 1. Asians (56.4%) constituted the largest ethnic group in the study, followed by Arabs (40.8%), Africans (3%), and Caucasians (3%). Most participants did not consume alcohol (97.2%) or smoke (75.7%). Time elapsed since T2DM diagnosis was as follows: ≤1 year, 29 patients (13.3%); 2–5 years, 66 (30.3%); 6–10 years, 67 (30.7%); and ≥ 11 years, 56 (25.7%).

**Table 1:** Baseline and anthropometric characteristics of study participants (*n* = 218)

Variable	Group	Frequency	Percentage
**Sex**	Male	186	85.3
	Female	32	14.7
**Ethnicity**	Arab	89	40.8
	Caucasian	3	1.4
	Asian	123	56.4
	African	3	1.4
**Alcohol intake**	Yes	6	2.8
	No	212	97.2
**Smoking**	Yes	53	24.3
	No	165	75.7
**Duration of diabetes**	≤1	29	13.3
**mellitus (year)**	2–5	66	30.3
	610	67	30.7
	≥11	56	25.7

### Clinical and biochemical characteristics of T2DM patients with confirmed or at high risk of ASCVD

The clinical and biochemical characteristics of the participants are presented in Table 2. The mean ± SD of age, height, weight, BMI, levels of HbA1c, eAG, FBS, TC, TGs, HDL-C, and LDL-C, systolic blood pressure (SBP), and diastolic blood pressure (DBP) were 50.8 ± 11.3 years, 168.8 ± 8.2 cm, 87.1 ± 17.6 kg, 30.6 ± 5.5 kg/m^2^, 8.3 ± 2%, 196.4 ± 86.3 mg/dL, 165.7 ± 68.5 mg/dL, 159.7 ± 79.8 mg/dL, 141.1 ± 100.2 mg/dL, 39.2 ± 11.6 mg/dL, 88.5 ± 54.6 mg/dL, 127.2 ± 16.8 mmHg, and 78.1 ± 9.7 mmHg, respectively. The median and range of the clinical and biochemical parameters are presented in Table 2.

**Table 2:** Clinical and biochemical characteristics of study participants (n = 218)

Parameter				
			All patients (***n*** = 218)	
	Mean	SD	Median	Range
**Age (years)**	50.8	11.3	51	78–29
**Height (cm)**	168.8	8.2	169	178–144
**Weight (kg)**	87.1	17.6	84.8	144–56.4
**BMI (kg/m**^**2**^**)**	30.6	5.5	29.9	51.8–20.8
**HbA1c (%)**	8.3	2	8	14.8–4.9
**eAG (mg/dL)**	196.4	86.3	184.3	1117–102.5
**FBS (mg/dL)**	165.7	68.5	144.3	414–48.6
**TC (mg/dL)**	159.7	79.8	164.5	680–61
**TG (mg/dL)**	141.1	100.2	149.5	636–46
**HDL-C (mg/dL)**	39.2	11.6	39	92–16
**LDL-C (mg/dL)**	88.5	54.6	85.5	234–12.8
**SBP (mmHg)**	127.2	16.8	125	180–90
**DBP (mmHg)**	78.1	9.7	80	110–60

*Abbreviations*: *BMI* Body mass index, *DBP* Diastolic blood pressure, *eAG* Estimated average glucose, *FBS* Fasting blood sugar, *HbA1c* Haemoglobin A1c, *HDL-C* High-density lipoprotein cholesterol, *LDL-C* Low-density lipoprotein cholesterol, *SBP* Systolic blood pressure, *TC* Total cholesterol, *TG* Triglyceride

### Assessment of physicians’ adherence to the guidelines of diabetic management in patients with confirmed or at high risk of ASCVD

The proportion of physicians’ adhering to the guidelines for the management of T2DM patients with either confirmed or at high risk of ASCVD is presented in Table 3. Among the 218 patients, 88 (44.4%) were prescribed SGLT2 inhibitors or GLP-1 receptor agonists and 34 (15.6%) were prescribed both SGLT2 inhibitors and GLP-1 receptor agonists. The overall adherence of physicians to the criterion for the management of T2DM patients with either confirmed or at high risk of ASCVD was 56% (95%CI: 49.3–62.6). The physicians in this study prescribed SGLT2 inhibitors to 109 patients (50%; 95%CI: 43.3–56.7) and a GLP-1 receptor agonist to 47 patients (21.6%; 95%CI: 16.1–27.1; Table 3). Empagliflozin (76.1%) and dapagliflozin (19.2%) were the most commonly prescribed SGLT2 inhibitors, and liraglutide (34%) and dulaglutide (23.4%) were the most frequently prescribed GLP-1 receptor agonists (Table 4). Dapagliflozin 5 mg/metformin 500 mg and insulin degludec 100 IU/mL/liraglutide were the least prescribed SGLT2 inhibitors and GLP-1 receptor agonists, respectively.

**Table 3:** Proportions of physicians adhering to the guidelines for diabetes management for patients with cardiovascular risk

Management of T2DM with cardiovascular risk	Proportion	95% CI
Prescription of SGLT2	109 (50%)	43.3–56.7
Prescription of GLP-1	47 (21.6%)	16.1–27.1
Overall adherence	122 (56%)	49.3–62.6

*Abbreviations*: *GLP-1* Glucagon-like peptide 1 agonist, *SGLT2* Sodium-glucose cotransporter 2 inhibitor

**Table 4:** Prescribed SGLT2 and GLP-1 medications for patients with diabetes mellitus and cardiovascular risk

SGLT2 (***n*** = 109)	F	%	GLP-1 (***n*** = 47)	F	%
Dapagliflozin	21	19.2	Dulaglutide	11	23.4
Dapaglifloxin 5 mg/ metformin 500 mg	2	18	Insulin degludec	2	4.3
Empagliflozin	83	76.1	Insulin degludec	5	10.6
Empagliflozin/metformin n HCL 12.5/1000 mg	3	2.8	Insulin glargine	4	8.5
–	–	–	Liragluide (Victoza)	16	34.04
–	–	–	Lixisenatide	5	10.6
–	–	–	Tresiba	4	8.5

*Abbreviations*: *F* Frequency, *GLP-1* Glucagon-like peptide 1 agonist, *SGLT2* Sodium-glucose cotransporter 2 inhibitor

### Assessment of physicians’ adherence to the guidelines based on biochemical markers

The level of physicians’ adherence, based on patients’ biochemical markers, is presented in Table 5. Significantly high levels of physicians’ adherence were observed in patients with very high levels of HbA1c (*P* < 0.001) and eAG (*P* = 0.002). This suggests that physicians are more likely to prescribe SGLT2 inhibitors and/or GLP-1 receptor agonists to patients with high levels of HbA1c and eAG. A similar pattern of physicians’ adherence to guidelines and the levels of FBS, SBP, TC, TG, LDL-C, and HDL-C and BMI was observed, although this was not significant.

**Table 5:** Distribution of physicians’ adherence according to different biochemical factors

Factor	Quartile	Adherence to guidelines	***P***-value
**HbA1c (%)**	<6.5	18 (39.1%)	<0.001
	6.5–7.5	20 (40.8%)	
	7.5<	84 (68.3%)	
**FBS (mg/dL)**	<100	8 (53.3%)	0.191
	100–125	21 (44.7%)	
	125<	93 (59.6%)	
**eAG (mg/dL)**	≤142	18 (37.5%)	0.002
	143–182	26 (50.0%)	
	≥183	78 (66.1%)	
**Systolic blood**	100–112	30 (55.6%)	0.66
**Pressure**	113–140	70 (54.3%)	
	141≤	22 (62.9%)	
**Diastolic blood**	≤70	42 (59.2%)	0.67
**Pressure**	71–80	55 (56.1%)	
	81≤	25 (51.0%)	
**TC (mg/dL)**	≤130	24 (54.5%)	0.970
	131–160	24 (57.1%)	
	161≤	74 (56.1%)	
**TG (mg/dL)**	≤83	11 (61.1%)	0.84
	84–140	39 (57.4%)	
	141≤	72 (54.5%)	
**LDL-C (mg/dL)**	50	10 (38.5%)	0.44
	51–86	47 (59.5%)	
	87≤	65 (57.5%)	
**HDL-C (mg/dL)**	≤33	31 (55.4%)	0.53
	34–46	55 (52.9%)	
	47≤	36 (62.1%)	
**BMI**	<25	12 (48%)	0.30
	25–29.9	43 (51.2%)	
	30≤	67 (61.5%)	

*Abbreviations*: *BMI* Body mass index, *eAG* Estimated average glucose, *DBP* Diastolic blood pressure, *FBS* Fasting blood sugar, *HbA1c* Haemoglobin A1c, *HDL-C* High-density lipoprotein cholesterol, *LDL-C* Low-density lipoprotein cholesterol, *SBP* Systolic blood pressure, *TC* Total cholesterol, *TG* Triglyceride

### Clinical and biochemical factors influencing physicians’ adherence to the guidelines of diabetes management in patients with T2DM with confirmed or at high risk of ASCVD

The results of the analysis of the logistic regression model applied to the patients’ anthropometric and biochemical characteristics are presented in Table 6. The odds ratios indicate the magnitude of association, whereas their corresponding *p* values indicate whether the association is significant. The univariate analysis showed that the physicians’ adherence to the guidelines was significantly correlated with the patients’ BMI (OR 1.08; 95% CI: 1.03–1.15) and levels of HbA1c (OR 1.31; 95% CI: 1.13–1.52) and eAG (OR 1.06; 95% CI: 1.03–1.12). Only the BMI and the levels of eAG and HbA1c were significantly associated with the physicians’ adherence to treatment guidelines, following a stepwise multivariate analysis. For every 1% increase in the level of HbA1c, 1 mg/dL increase in the level of eAG, and 1 unit increase in BMI, the physicians’ adherence increased by 55, 2.5, and 9.3%, respectively.

**Table 6:** Univariate and multivariate logistic regression analyses of factors associated with physicians’ adherence to diabetes guidelines

Factor			Physicians’ adherence to guidelines					
		Univariate				Multivariate		
	OR	95% CI		***P*** value	OR	95% CI		***P*** value
Sex (female)	1.18	0.55	2.52	0.67	–	–	–	–
Alcohol intake	0.15	0.02	1.31	0.08	–	–	–	–
Smoking	1.57	0.83	2.97	0.17	–	–	–	–
**Duration of diabetes mellitus (year)**	–	–	–	–	–	–	–	–
2–5	1.07	0.45	2.57	0.88	–	–	–	–
6–10	1.3	0.55	3.16	0.53	–	–	–	–
≥ 11	2.26	0.90	5.67	0.08	–	–	–	–
Age	0.99	0.97	1.02	0.79	–	–	–	–
BMI	1.08	1.03	1.15	0.004	1.093	1.003	1.191	0.043
HbA1c (%)	1.31	1.13	1.52	<0.001	1.545	1.197	1.994	0.001
eAG (mg/dL)	1.06	1.03	1.12	0.001	1.025	1.010	1.040	0.001
FBS (mg/dL)	1.01	1	1.012	0.06	–	–	–	–
TC (mg/dL)	1.004	0.99	1.01	0.19	–	–	–	–
TG (mg/dL)	1.001	0.99	1.004	0.60	–	–	–	–
HDL-C (mg/dL)	1.01	0.98	1.04	0.51	–	–	–	–
LDL-C (mg/dL)	1.007	0.99	1.02	0.14	–	–	–	–
SBP (mmHg)	1.002	0.98	1.02	0.82	–	–	–	–
DBP (mmHg)	1.002	0.97	1.03	0.87	–	–	–	–

*Abbreviations*: *BMI* Body mass index, *DBP* Diastolic blood pressure, *eAG* Estimated average glucose, *FBS* Fasting blood sugar, *HbA1c* Haemoglobin A1c, *HDL-C* High-density lipoprotein cholesterol, *LDL-C* Low-density lipoprotein cholesterol, *SBP* Systolic blood pressure, *TC* Total cholesterol, *TG* Triglyceride

## Discussion

Physicians’ adherence to evidence-based treatment guidelines for various illnesses, barriers to physicians’ adherence, and ways to overcome these barriers have previously been investigated. In Cyprus, adherence to clinical guidelines by physicians in the management of hypertension is inconsistent [12]. Although physicians were aware of the clinical guidelines for the management of hypertension, more than a quarter of high-risk patients remained untreated, while 40% of low-risk patients were prescribed inappropriate treatment. Poor adherence to the 2007 ATS/IDSA guidelines for treating pulmonary non-tuberculous mycobacterial disease [13] and common prescriptions with sub-optimal or potentially harmful treatments have also been reported, highlighting the need for the consistent adherence of physicians to treatment guidelines [14]. Physicians’ adherence is essential when implementing evidence-based guidelines. Therefore, modification of evidence-based guidelines to suit the local or regional conditions is necessary [15].

In this study, most patients with T2DM had various risk factors. The mean HbA1c level was 8.3%, which is higher than the recommended HbA1c target of <7%. Therefore, the overall HbA1c level in our study was sub-optimal. In a similar study, most of the most health care providers that participated (69.9%) were confident in managing these patients, whereas 79.2% were aware of the accessibility to local guidelines [15]. One-fifth of the participating physicians managed patients by targeting an HbA1c level of <6.5%. However, 3.3% of the physicians were content with maintaining an HbA1c level of up to 8.0% [15].

GLP-1 receptor agonists and SGLT2 inhibitors have cardiovascular benefits in patients with T2DM and ASCVD [16]. There was a high adherence of physicians to evidence-based guidelines in our study; fifty-six percentage of the selected physicians had prescribed SGLT2 inhibitors and GLP-1 agonists while managing patients with T2DM, who either developed or were at high risk of developing CVDs. GLP-1 receptor agonists are efficacious for patients with confirmed ASCVD [16]. The REWIND cardiovascular trial reported that administration of dulaglutide caused a considerable decline in major adverse cardiovascular events, such as composite outcome (cardiovascular death and/or end-stage renal disease) and non-fatal myocardial infarction or non-fatal stroke [17]. The cardiovascular effects of GLP-1 receptor agonists are mediated by lowering blood pressure, body weight, LDL cholesterol levels, and glucose, in addition to the elimination of low-grade inflammation, vasodilation, and natriuresis [15].

The SUSTAIN 6 cardiovascular outcomes trial assessed the cardiovascular safety of semaglutide in patients with T2DM and associated cardiovascular defects. This 2-year trial reported that semaglutide significantly reduced the risk of developing a three-component MACE endpoint namely cardiovascular death, non-fatal stroke, and non-fatal heart attack. The relative reduction of MACE risk was 26% when compared with that of the placebo (HR 0.74; 95% CI: 0.58–0.95; *P* < 0.001 for non-inferiority), over a median observation time of 2.1 years, and a primary composite outcome of 6.6% was observed for patients treated with semaglutide versus 8.9% for the placebo [18, 19]. This was consistent with the findings of this study, in which liraglutide, followed by dulaglutide were the most frequently prescribed GLP-1 receptor agonists. Among the SGLT2 inhibitors, empagliflozin has a slightly higher cardiovascular benefit for patients with confirmed ASCVD [16]. The results of this study also showed that empagliflozin combined with dapagliflozin are the most frequently prescribed SGLT2 inhibitors.

Although we observed high adherence of physicians to the 2018 ADA and European Association guidelines designed for the management of patients with T2DM and ASCVD, there remains scope for improvement. Previous studies have explored the benefits of managing modifiable ASCVD risk factors in patients with diabetes and reported a considerable reduction in deaths and ASCVD events and improvement in long-term benefits [5, 6]. Therefore, the use of evidence-based approaches for the management of patients with T2DM and ASCVD is highly recommended [7].

This study has some limitations. The number of patients included in the analysis was small. The study was cross-sectional in nature and was conducted in a single hospital setting. Hence, the findings cannot be generalised to a wider range of adult T2DM population in the UAE, and therefore further studies are warranted.

## Conclusions

Evaluating the adherence of physicians to evidence-based treatment guidelines for the management of T2DM patients with ASCVD is crucial to improve overall care. Based on the treatment guideline: weak recommendation for starting SGLT2 inhibitors and GLP-1 receptor agonists for patients with established CVD or CKD, this study showed that a moderate proportion of the physicians (56%) adhered to the guidelines for diabetes management for patients with ASCVD because the prescription of SGLT2 inhibitors and GLP-1 receptor agonists are not strongly recommended. We also found significantly higher levels of physicians’ adherence to the guidelines in patients with high levels of HbA1c (>7.5) and eAG (≥183). The adherence level of physicians to the treatment guidelines was significantly associated with BMI and levels of HbA1c and eAG. This implies the SGLT2 inhibitors and GLP-1 receptor agonists were prescribed for cases with clear manifestations of T2DM symptoms. The data generated here can help healthcare workers and policy makers in the UAE to make well-informed decisions regarding diabetes care and strategies for preventing complications and identifying risk factors associated with T2DM.

## Data Availability

All data generated or analysed during this study are included in this published article.

## Abbreviations

ADA: American Diabetes Association
ASCVD: Atherosclerotic cardiovascular disease
BMI: Body mass index
CKD: Chronic kidney disease
DCCPG: Diabetes Canada Clinical Practice Guidelines
eAG: Estimated average glucose
FBS: Fasting blood sugar
GLP-1: Glucagon-like peptide 1
HbA1c: Hemoglobin A1c
HDL-C: High-density lipoprotein cholesterol
IDF: International Diabetes Federation
LDL-C: Low-density lipoprotein cholesterol
SGLT2: Sodium-glucose co-transporter-2
T2DM: Type 2 diabetes mellitus
TC: Total cholesterol
TG: Triglyceride

## References

[1] NCD Risk Factor Collaboration (NCD-RisC) (2016). Worldwide trends in diabetes since 1980: a pooled analysis of 751 population-based studies with 4.4 million participants. Lancet..

[2] International Diabetes Federation (2009). IDF Diabetes Atlas.

[3] Wang CC, Shah AC, Alexandria VA (2017). Medical management of type 1 diabetes.

[4] American Diabetes Association (2018). Cardiovascular disease and risk management: Standards of medical care in diabetes - 2018. Diabetes Care.

[5] Gæde P, Oellgaard J, Carstensen B, Rossing P, Lund-Andersen H, Parving HH, Pedersen O (2016). Years of life gained by multifactorial intervention in patients with type 2 diabetes mellitus and microalbuminuria: 21 years follow-up on the Steno-2 randomised trial. Diabetologia..

[6] Khunti K, Kosiborod M, Ray KK (2018). Legacy benefits of blood glucose, blood pressure and lipid control in individuals with diabetes and cardiovascular disease: time to overcome multifactorial therapeutic inertia?. Diabetes Obes Metab.

[7] Gregg EW, Sattar N, Ali MK (2016). The changing face of diabetes complications. Lancet Diabetes Endocrinol.

[8] Li R, Zhang P, Barker LE, Chowdhury FM, Zhang X (2010). Cost-effectiveness of interventions to prevent and control diabetes mellitus: a systematic review. Diabetes Care.

[9] Burant CF, Young LA, Alexandria VA (2012). Medical management of type 2 diabetes.

[10] Diabetes Canada Clinical Practice Guidelines Expert Committee (2018). Diabetes Canada 2018 Clinical Practice Guidelines for the Prevention and Management of Diabetes in Canada. Can J Diabetes.

[11] Zhu Y, Xu J, Zhang D, Mu X, Shi Y, Chen S, et al. Efficacy and Safety of GLP-1 Receptor Agonists in Patients With Type 2 Diabetes Mellitus and Non-Alcoholic Fatty Liver Disease: A Systematic Review and Meta-Analysis. Front Endocrinol. 2021:1–13. 10.3389/fendo.2021.769069. 10.3389/fendo.2021.769069 PMC8696030 34956080

[12] Theodorou M, Stafylas P, Kourlaba G, Kaitelidou D, Maniadakis N, Papademetriou V. Physicians' perceptions and adherence to guidelines for the management of hypertension: a national, multicentre, prospective study. Int. J Hypertens. 2012;2012:503821. 10.1155/2012/503821 PMC3515898 23251788

[13] Adjemian J, Prevots DR, Gallagher J, Heap K, Gupta R, Griffith D (2014). Lack of adherence to evidence-based treatment guidelines for nontuberculous mycobacterial lung disease. Ann Am Thorac.

[14] Goldberg SL, Akard LP, Dugan MJ, Faderl S, Pecora AL (2015). Barriers to physician adherence to evidence-based monitoring guidelines in chronic myelogenous leukemia. J Oncol Pract.

[15] Savona-Ventura C, Vassallo J (2019). MGSD-Education Study Group. Healthcare professionals’ perceptions of type 2 diabetes mellitus care in the Mediterranean region. Diabetes. Therapy..

[16] Davies MJ, D'Alessio DA, Fradkin J, Kernan WN, Mathieu C, Mingrone G, Rossing P, Tsapas A, Wexler DJ, Buse JB, Management of hyperglycaemia in type 2 diabetes (2018). A consensus report by the American Diabetes Association (ADA) and the European Association for the Study of Diabetes (EASD). Diabetologia..

[17] Gerstein HC, Colhoun HM, Dagenais GR, Diaz R, Lakshmanan M, Pais P, Probstfield J, Riesmeyer JS, Riddle MC, Rydén L, Xavier D, Atisso CM, Dyal L, Hall S, Rao-Melacini P, Wong G, Avezum A, Basile J, Chung N, Conget I, Cushman WC, Franek E, Hancu N, Hanefeld M, Holt S, Jansky P, Keltai M, Lanas F, Leiter LA, Lopez-Jaramillo P, Cardona Munoz EG, Pirags V, Pogosova N, Raubenheimer PJ, Shaw JE, Sheu WH, Temelkova-Kurktschiev T (2019). Dulaglutide and cardiovascular outcomes in type 2 diabetes (REWIND): a double-blind, randomised placebo-controlled trial. Lancet..

[18] Marso SP, Bain SC, Consoli A, Eliaschewitz FG, Jódar E, Leiter LA, Lingvay I, Rosenstock J, Seufert J, Warren ML, Woo V, Hansen O, Holst AG, Pettersson J, Vilsbøll T (2016). Semaglutide and cardiovascular outcomes in patients with type 2 diabetes. N Engl J Med.

[19] American Diabetes Association (2016). Cardiovascular disease and risk management. Diabetes Care.

